# The Use of Dried Milk

**Published:** 1914-08

**Authors:** 


**The Use of Dried Milk.** Nash, in Pediatrics, May, 1914, advises the use of milk dried upon cylinders quickly at a temperature of 250 F., according to the process of Just and Hatmaker, as he has seen good results from this food.—C. G. L-W.

